# Timeliness of immunisation with the pentavalent vaccine at different levels of the health care system in the Lao People’s Democratic Republic: A cross-sectional study

**DOI:** 10.1371/journal.pone.0242502

**Published:** 2020-12-08

**Authors:** Lisa Hefele, Sengdavanh Syphan, Dalouny Xayavong, Anousin Homsana, Daria Kleine, Phetsavanh Chanthavilay, Phonethipsavanh Nouanthong, Kinnaly Xaydalasouk, Outavong Phathammavong, Somxay Billamay, Anonh Xeuatvongsa, Daniel Reinharz, Antony P. Black, Claude P. Muller

**Affiliations:** 1 Lao-Lux Laboratory, Institut Pasteur du Laos, Vientiane, Lao PDR; 2 Department of Infection and Immunity, Luxembourg Institute of Health, Esch-sur-Alzette, Grand-Duchy of Luxembourg; 3 Lao Tropical and Public Health Institute, Vientiane, Lao PDR; 4 Luxembourg Development Cooperation Agency, Vientiane, Lao PDR; 5 Children Hospital, Vientiane, Lao PDR; 6 Expanded Programme on Immunisation, Vientiane, Lao People’s Democratic Republic; 7 Département de Médecine Sociale et Préventive, Université Laval, Québec, Canada; Charite Universitatsmedizin Berlin, GERMANY

## Abstract

**Background:**

The timely administration of vaccines is considered to be important for both individual and herd immunity. In this study, we investigated the timeliness of the diphtheria-tetanus-whole cell pertussis-hepatitis B-*Haemophilus influenzae* type b (pentavalent) vaccine, scheduled at 6, 10 and 14 weeks of age in the Lao People’s Democratic Republic. We also investigated factors associated with delayed immunization.

**Methods:**

1162 children aged 8–28 months who had received the full course of the pentavalent vaccine at different levels of the health care system were enrolled. Vaccination dates documented in hospital records and/or immunisation cards were recorded. Age at vaccination and time intervals between doses were calculated. Predictors for timely completion with the pentavalent vaccine at 24 weeks were assessed by bivariate and multivariable analyses.

**Results:**

Several discrepancies in dates between vaccination documents were observed. In general, vaccination with the pentavalent vaccine was found to be delayed, especially in health care settings below the provincial hospital level. Compared to the central hospital level, less participants who were vaccinated at the district/health center level received the third dose by 16 (48% at the central hospital level vs. 7.1% at the district and 12.4% at the health center level) and 24 weeks of age (94.4% at the central hospital level vs 64.6% at the district-outreach and 57.4% at the health center level) respectively. In logistic regression analyses, lower education level of the mother as well as vaccination by outreach service, were independently associated with delayed completion of vaccination.

**Conclusion:**

We observed a general delay of vaccination, especially at lower ranked facilities, which correlated with indicators of poor access to health services. This highlights the need for further improving health equity in rural areas. Age-appropriate vaccination should become a quality indicator for the national immunization programme. In addition, we recommend further training of the health care staff regarding the importance of reliable documentation of dates.

## Introduction

The timely administration of vaccines is considered to be important for both individual and community immunity. Studies in the UK [1], China [2], the USA [3] and lower-income countries such as Senegal [4], Sri Lanka [5], Malawi [6] and India [7, 8] have investigated the impact of timeliness on vaccination. Delayed vaccinations of children may increase the risk of infection before vaccination, compromising the success of the intervention as well as herd immunity. On the other hand, vaccinations given too early or without sufficient interval between the doses may not be fully protective [1].

In the Lao People´s Democratic Republic (PDR), the expanded programme on immunization (EPI) is one of the most successful public health programmes and routine vaccination coverage has increased for more than ten years. Currently, the National Immunization Programme in Lao PDR includes vaccines against tuberculosis, measles, rubella, diphtheria, tetanus, pertussis, *H*. *influenzae* type b, polio, pneumococcal disease and Japanese encephalitis. Vaccination of children with vaccines included in the National Immunization Program is free of charge.

The estimated coverage for the 3^rd^ dose of the pentavalent diphtheria-tetanus-pertussis-hepatitis B-*Haemophilus influenzae* type b vaccine (pentavalent vaccine) was 84% [9] nationwide in 2018 and 84.1% specifically in Bolikhamxay province in 2017 [10]. However, it was reported in 2012 that only a small number of children had received all 3 doses of the pentavalent vaccine by the recommended age of 4 months in Lao PDR [11]. While there are clear national recommendations for delivery of the pentavalent vaccine doses in Lao PDR, little is known at which age children actually receive the vaccine within the National Immunization Program and whether this may impact the immune response. The pentavalent vaccine is scheduled at 6, 10 and 14 weeks of age. Vaccination schedules vary between countries. Vaccination with the DTP-containing vaccine in Thailand follows a 2-4-6 months schedule with a booster vaccination at 4 years [12]. The United States of America follow the same schedule for the primary vaccination but include a booster at 15 months [13] while some European countries such as Germany, Luxembourg and Belgium follow a 2-3-4 months schedule but differ with respect to the timing of the booster dose [14]. Generally, the minimum age of vaccination for the DTP-containing vaccine is six weeks and the recommended spacing between the doses is four weeks. Vaccination doses given too early after the first one may result in an impaired vaccine response [13]. However, there is no consensus on definitions for vaccination timeliness [15].

Achieving a high coverage with the (monovalent) hepatitis B birth dose is important to prevent mother-to-child transmission of Hepatitis B. Even though the birth dose was introduced in Lao PDR in 2003, the nationwide coverage was only 55% in 2018 [16]. Therefore, the birth dose coverage continues to be an important issue in Lao PDR.

In Lao PDR, central hospitals (CH) in Vientiane Capital represent the highest level of the health care system, followed by provincial hospitals (PH), district hospitals (DH) and health centers (HC) [17]. CHs are tertiary care facilities, PHs and DHs provide health promotion, disease prevention and treatment services, but are limited in capacity and expertise. HCs provide only basic medical services, including immunization services and maternal, new-born and child health services [18, 19]. For vaccinations, the parents tend to rely on the nearest health care facility. Although vaccination for children is free, not all families can afford out-of-pocket payments for travel and sustain the loss of a day's work. Typically, health care facilities (HCF) such as HC or DH provide immunization services to several villages within a flexible radius. Vaccinations are registered in the yellow child vaccination card, which stays with the family, and the hospital records, which can consist of one registry book at the mother and child department but also includes the vaccination books of the EPI team, typically one vaccination book for each village. Regardless of where the vaccination takes place, the children are usually listed in the book of the home village and in case of outreach vaccination, the books are taken to the villages. The hospital records as well as vaccination cards are standardized and provided by the health offices.

The primary objective of this study was to investigate the timeliness of vaccination with the three doses of the pentavalent routine childhood vaccine in fully vaccinated children in Bolikhamxay province and Vientiane Capital. In addition, the timeliness of Hepatitis B birth dose was assessed. For this purpose, the dates of vaccination as recorded in the vaccination card and hospital records were compared and the proportion of children that received the vaccination with delay was estimated. Furthermore, we investigated risk factors associated with delayed vaccination.

## Participants and methods

### Participants

The study took place in the context of a larger vaccine immunogenicity study in Bolikhamxay province and in Vientiane capital in 2017/18 (see S1 File) [20]. All participants had received the full course of the pentavalent vaccine, documented in either the hospital records or vaccination card. In Vientiane Capital, 319 children aged 8 to 23 months and their parents/guardians who visited the Children´s Hospital for unrelated health reasons or Measles and Rubella vaccination were enrolled. Bolikhamxay is a central province only about 150 km away from the capital, on the highway to the South. Bolikhamxay comprises 291 villages with 53 964 households including 304 000 inhabitants in 7 districts [21]. In Bolikhamxay province, 843 children aged 8 to 28 months were recruited, who were vaccinated in the PH, three DHs and ten HCs. Before the start of the sample collection, the study was explained to the head of the participating village and to the parents of the participants by a health care worker. All parents/guardians signed the informed consent form and could withdraw their participation at any time. A standardized questionnaire was designed to collect information about the participant´s socio-economic background, access to health care, history and location of vaccination. The detailed information collected by the questionnaire can be found in S1 Table in S1 File of the previously published study. Vaccination histories were verified and confirmed in the hospital records at the HCF, if available, as well as on the vaccination card. The data was double entered into Epidata [22] independently before data analysis. Serum samples were collected from participating children to assess antibody levels. The study was approved by the Lao National Ethics Committee (Reference numbers 033/2017/NECHR, 032/2017/NECHR, 031/2017/NECHR, 056/2017/NECHR) and by the internal ethics review board of the Institut Pasteur du Laos.

### Vaccination dates

During recent years, the vaccination schedule expanded with the inclusion of new vaccines and/or additional doses, and the layout of vaccination cards and hospital records were gradually adapted. During the review of the vaccination history, we came across different formats and versions of the vaccination card and hospital records. Major changes in format were introduced with the pneumococcal vaccine (PCV13), the inactivated polio vaccine (IPV), the Japanese encephalitis vaccine (JEV) and the second dose of the measles-rubella vaccine (MR). Not all facilities used the latest date version of the hospital records or vaccination cards. However, since the pentavalent vaccine was introduced already in 2009/10, it was included in all versions of the vaccination card and the hospital records inspected in this study [23].

The vaccination history of the participants was recorded from the hospital records and/or vaccination cards. The age of the participants in weeks at the time of the vaccination with the pentavalent vaccine and with the hepatitis B birth dose was calculated based on the birthdate and the date of the vaccination. Since there is no consensus-definition of “timely vaccination” in lower middle-income countries, as discussed in a review [15], we used both continuous and categorical measures. The age when receiving each vaccine dose was defined as: “timely” when between 6–7 weeks for dose 1 (pentavalent 1), 10–11 weeks for dose 2 (pentavalent 2) and 14–15 weeks for dose 3 (pentavalent 3) (Table 1). When the participant was older or younger, the vaccination was considered to be “earlier” or “later” than recommended. An interval of 4 weeks is recommended between each dose and intervals shorter than 4 weeks or longer than 5 weeks were considered “shorter” or “longer” than recommended. The National Immunization Programme of the Lao PDR recommends the primary vaccination with the DTP-containing vaccine is completed at 14 weeks and the WHO guidelines specify completing the vaccination course latest at 6 months (24 weeks) of age [24]. In this study, we considered 16 weeks (in order to give some time margin) and 24 weeks (the latest WHO recommendation for completeness) as cut-off for”early timely” and “late timely” completion of vaccination with the pentavalent vaccine (Table 1). The proportion of participants that had received the pentavalent vaccine by 16 and 24 weeks at each health care level was compared to the level of the CH. Furthermore, we used 24 weeks as a cut-off in order to identify factors associated with the timely completion of the schedule or not.

**Table 1:** Definitions for timeliness of vaccination with the pentavalent vaccine.

Vaccine dose	Recommended age	Definition of timeliness	Explanation
Pentavalent 1	6 weeks	6–7 weeks	Adherence to vaccination schedule
Pentavalent 2	10 weeks	10–11 weeks	Adherence to vaccination schedule
Pentavalent 3	14 weeks	14–15 weeks	Adherence to vaccination schedule
		by 16 weeks	Early completion of full vaccination with the pentavalent vaccine
		by 6 months	Late completion of full vaccination with the pentavalent vaccine

In order to detect irregularities in the vaccination documents of participants who had both the hospital records and vaccination card available, the time discrepancy between documented vaccination dates was obtained by subtracting the vaccination date in the vaccination card from the vaccination date in the hospital records. Vaccination dates in the vaccination cards were considered more reliable as the cards stay with the mothers and are filled in on the day of the vaccination. Hospital records can also be entered at a later time point and problems with medical documentation has been reported in Lao PDR before [25]. Therefore, we gave priority to the vaccination cards to calculate the median age at vaccination and the intervals between vaccinations. Whenever the vaccination card was not available, the date in the hospital records was used.

### Laboratory analysis

The serum samples were analysed by commercial ELISA kits as described elsewhere [20]. Protective immunity was considered if the participants had an anti-diphtheria titer ≥0.1 IU/ml, an anti-tetanus titer >0.5 IU/ml, an anti-hepatitis B titer >10 IU/L and an anti-*Haemophilus influenzae* type b (Hib) titer >1.0μg/ml. A titer ≥22 U/ml was used as indication of exposure to the vaccine antigen for *B*. *pertussis*.

### Statistical analysis

Data analyses were conducted using R software (version 3.5.3) [26] with the following packages: “tidyverse” [27], “lubridate” [28], “MASS” [29], “rcompanion” [30], “lmtest” [31], “car” [32], “epitools” [33], “ggplot2” [34], “survival” [35], “survminer” [36] and “pROC” [37].

Survival analysis by the Kaplan-Meier method was performed to present timeliness of vaccination for each of the three doses of pentavalent vaccine at any given age (S3 Fig), a method suggested by Lauberau et al. (2002) [38]. For the Kaplan-Meier curve, we used the age at vaccination as based on the date in the vaccination card, and if there was no card available, we used the date in the hospital records. Participants for which the calculated age at vaccination was negative (date of vaccination before date of birth, a documentation error) were excluded.

In order to assess whether any of the socio-demographic or vaccination related factors are associated with the completion of primary vaccination with the pentavalent vaccine by 24 weeks (6 months) of age, Chi‐square test and Fisher’s exact test were performed. Odds ratio (OR), 95% confidence intervals (CI) and p-value were calculated. We performed logistic regression analysis in order to investigate the association between the binomial response variable (completion of vaccination by 24 weeks) and socio-economic or vaccination-related factors. Only variables with p-values <0.2 were included in the generalized linear models (GLMs). The correlation (correlation value >0.5) and/or multicollinearity (variance inflation factor >2–5) of independent variables was checked, and in that event, the variable which was considered to be less important and/or with the lower impact was not included. We performed binary logistic regressions using a stepwise method for removing variables that are not associated with the response variable one by one, while considering both the p-value of the variable and the Akaike Information Criterion. The models were tested for possible interactions. The best fitting model was selected by comparing the AIC weights for a set of fitted models. The final model was assessed in comparison with the null model using a likelihood ratio test. In addition, the individual association of the variables in the model was tested by Wald tests. In order to assess the predictive ability of the model, the area under the curve (AUC) was calculated using the “roc” R function. A p-value <0.05 was considered statistically significant.

## Results

### Documentation of vaccination

At the CH, the three doses of the pentavalent vaccine were verified in the vaccination card of all participants (100%) from Vientiane. Most of these children (65.5%) were vaccinated with all three doses at the Children´s hospital, the others received some or all of the doses at another CH in Vientiane. In Bolikhamxay, the parents of 620 children (73.6%) presented the vaccination card. The vaccination entries in the hospital records were found for 83.3% of the children and for 56.8%, both sources were available.

### Comparison of vaccination cards and hospital records

At the Children’s hospital, it was possible to review the hospital records of all three doses for 88 (27.6%) participants, and for at least one dose for 155 (48.6%) participants. All vaccination dates matched between the vaccination card and hospital records. However, for 8 (2.5%) participants, the birth date entry in vaccination card and hospital records did not match.

In Bolikhamxay, both hospital records and vaccination cards were available from 479 (56.8%) participants. We discovered a number of discrepancies between the two documents. The birth dates of 34 (7.1%) participants differed between vaccination card or hospital records. From the 479 participants in Bolikhamxay with both hospital records and vaccination card, 180 (37.6%) had at least one discrepancy or mismatch between the hospital records & vaccination card date-pairs of at least one of the three doses. From these 180 participants, 43.3% had a mismatch in the dates for pentavalent 1 and 62.8% for pentavalent 3. 17.2% had mismatches for all three date-pairs. The date for pentavalent 3 in the hospital records was before the vaccination card in 71 (39.4%) cases while it was later in only 37 (20.6%) of the 180 participants. The proportion of mismatches increased from pentavalent 1 to 3. At the PH level, only 2.4% to 6.0% of the vaccination dates mismatched from pentavalent 1 to 3 (Table 2). At the DH and HC level, there was a higher proportion of mismatches between vaccination dates at the facility as compared to outreach. The highest proportion of mismatches (41.1%) was found at the health center level among the participants vaccinated directly at the facility. Here, also the largest mean discrepancy between the vaccination card and hospital records was also observed (-9.9 days) (S1 Table in S1 File).

**Table 2:** Comparison between vaccination date pairs from the vaccination books and vaccination cards.

	Provincial hospital		District hospitals				Health center			
			Facility		Outreach		Facility		Outreach	
	n mismatch* /N	%	n mismatch /N	%	n mismatch /N	%	n mismatch /N	%	n mismatch /N	%
pentavalent 1	2/83	2.41	16/63	25.40	5/45	11.11	11/58	18.97	44/230	19.13
pentavalent 2	2/83	2.41	17/62	27.42	6/45	13.33	20/58	35.09	50/232	21.55
pentavalent 3	5/83	6.02	19/60	31.67	6/46	13.04	23/56	41.07	60/234	25.64

N = total number per group (in some instances, the dates were unreadable or missing and only the signature of the health care worker was present); pentavalent = Diphtheria-Tetanus-Pertussis whole cell–Hepatitis B–*Haemophilus influenzae* Type B vaccine

* Mismatch = The date for the specific dose of the pentavalent vaccine in the hospital records and vaccination cards did not match

Several other observations were made: In some cases (7.8%) among the 180 participants with both records, either pentavalent 2 or 3 or both, the hospital records had the same entry as pentavalent 1 and/or 2 in vaccination card; many of the mismatches differed by less than 7 days for at least 1 date-pair (23.9%); for 9.4% of participants all dates in the hospital records were before the vaccination card; for 7.2%, both the intervals were 4 weeks in the hospital records but not in the vaccination card and at least one of the dates in the hospital records was before the one in the vaccination card. On very few occasions, two doses were denoted with the same date or an obvious mistake was made with respect to the recorded year or the day. Because the vaccination card were less likely to be tampered with, the dates in the vaccination card were given priority in case of mismatches.

### Age at vaccination

The median age at vaccination with pentavalent dose 1, 2 and 3, and therefore also the difference between the median age and the recommended age, increased at the lower ranked health care facilities (Table 3) and with each dose, e.g. the median age at the first dose was 6.7 weeks at the central hospital level, but 9.4 weeks at the health center level when participants were vaccinated by outreach services.

**Table 3:** Median age at vaccination in weeks according to health care level.

	Median (Interquartile range) age at vaccination^a^		
	pentavalent 1	pentavalent 2	pentavalent3
Health care level	recommended at 6 weeks	recommended at 10 weeks	recommended at 14 weeks
CH	6.71 (6.43–7.00)	11.43 (11.14–12.14)	16.14 (15.71–17.29)
PH	6.86 (6.71–7.43)	11.57 (11.14–12.71)	16.29 (15.71–17.89)
DH—facility	7.14 (6.71–7.71)	12.21 (11.71–13.57)	18.00 (16.86–19.96)
DH—outreach	8.5 (7.04–10.21)	13.57 (12.14–18.36)	20.14 (17.64–28.25)
HC—facility	7.14 (6.64–9.36)	13.14 (11.46–16.82)	19.71 (16.79–23.61)
HC—outreach	9.36 (7.29–12.14)	15.64 (12.68–20.86)	22.86 (19.00–29.71)

^a^The age was calculated with the date written in the YC, if the YC was not present, the date in the HR was used

Pentavalent = Diphtheria-Tetanus-Pertussis whole cell–Hepatitis B–Haemophilus influenzae Type B vaccine, CH = central hospitals, PH = Provincial hospital, DH = District hospitals, HC = Health centers

The Kaplan-Meier curve (S1 Fig) shows that at the CH level, 50% of the children were vaccinated at the age of 6.7 weeks with pentavalent 1 compared to almost 9 weeks at the HC level. For pentavalent 3, there was a considerable delay at all lower ranked facilities. At the HC outreach level, 50% vaccination coverage was reached at 23.1 weeks (CI: 22.4–24.4) and considerably fewer children were vaccinated by 24 weeks (6 months) of age compared to CH, PH and DH levels (S1 Fig).

**Fig 1:**
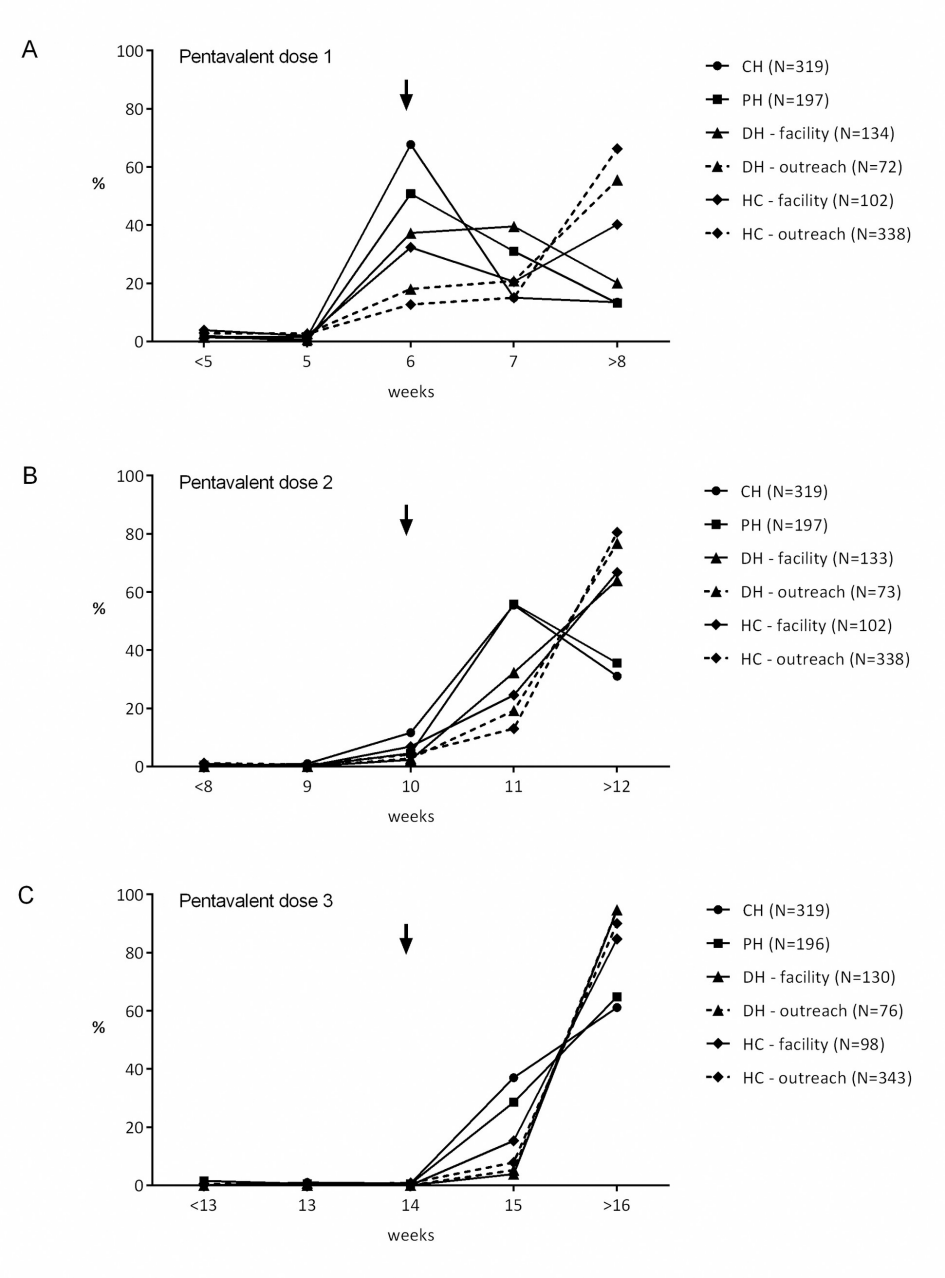
Proportion of participants vaccinated with pentavalent 1, 2 and 3 at a given age by health care levels. Arrows indicate recommended week of vaccination. CH = central hospital level, DH = hospital level, HC = health center level. The age at vaccination was calculated based on the vaccination card, and in case the vaccination card was not available, the date in the hospital records was used.

In order to estimate the timeliness of vaccination, the proportion of participants vaccinated at a given age was calculated (Fig 1). The proportion vaccinated at the recommended 6 weeks of age decreased from 67.7% at the CH to 32.4% at the HC, and outreach vaccination was as low as 12.7%. For pentavalent 2, most of the participants were still vaccinated within a week of delay at the CH and PH (55.5% to 55.8%, respectively). The majority of participants (ranging from 61.1% at the CH to 94.7% for DH outreach) were vaccinated with a delay of 2 or more weeks for pentavalent 3 at all locations.

Compared to the central hospital level, fewer participants who were vaccinated at the district and health center level received the third dose by 16 weeks (48% (95% CI [42.6–53.6]) at the central hospital level vs. 7.1% (95% CI [3.4–10.8]) at the district and 12.4% (95% CI [9.2–15.5] at the health center level) (S2 Fig). However, by week 24, more than 90% of the participants at the CH and PH level (94.7% (95% CI [92.2–97.1]) and 92.6% (95% CI [88.8–96.3]) respectively), as well as participants vaccinated directly at the DH level (91.5% (95% CI [86.5–96.6])) had received pentavalent 3. Fewer participants that were vaccinated at the HC level and during DH outreach services were vaccinated with the 3^rd^ dose by 24 weeks of age (58.4% (95%CI [54–62.7], p<0.0001) (S2 Fig) when compared to the CH.

In order to investigate any association between delayed vaccination (defined as age at pentavalent 3 >16 weeks) and seroprotection against any of the five vaccine components, bivariate analyses were performed among those children vaccinated at the CH and PH level. Receiving the 3^rd^ (last) dose of the pentavalent vaccination more than 16 weeks after birth was not associated with lower seroprotection (S2 Table in S1 File) in this study.

### Interval between vaccination doses

At the CH level, the median interval between pentavalent dose 1 and 2 was 4.7 weeks (S3 Table in S1 File; S3 Fig) and only somewhat higher for outreach vaccination at the HC and DH level (5.1 and 5.4 weeks respectively). At the CH and PH level, 62–70% were vaccinated within the recommended interval of 4 weeks between dose 1 and 2 and dose 2 and 3. Considerably less participants were vaccinated within 4-week intervals in lower ranked facilities. Only very few participants were vaccinated within less than 4 weeks (rates ranging from 0.0% to 4.4%) at any health care level.

For most participants at the district and health center level, at least one of the two intervals (pentavalent 1 and 2 and/or pentavalent 2 and 3) was longer than 4 weeks (ranging from 57.1% to 83.2%) (S4 Table in S1 File).

### Predictors for timely completion of vaccination with the pentavalent vaccine

We used timely completion with pentavalent 3 at 24 weeks as binary outcome and performed logistic regression analyses to identify predictors in Bolikhamxay province. The bivariate analyses (S5 Table in S1 File) showed that being an older mother with a higher education and a higher household income was more often associated with timely vaccination. Markers of inequity in access to health care (delivery at home, no ANC and no hepatitis B birth dose) all were also predictors of delayed vaccinations. Travel time to health care facilities, vaccination by outreach and not belonging to the Tai-Kadai ethnic group emerged as obstacles to timely vaccination.

In logistic regression analyses all variables associated with the outcome with a p<0.2 were included. However, since “place of birth” and “district” correlated with the “place of vaccination”, these variables were excluded from analyses. “Travel time to the next health care facility” was used as surrogate for distance and “received ANC” was used as surrogate for ANC practices in the modelling. The variables “age of participant”, “age of mother”, “number of household members”, “number of siblings” and “travel time to the next health care facility” were included in the logistic model as numeric variables. Several variables remained independently associated with the outcome (Table 4). Participants were more likely to have completed the primary vaccination series by the age of 24 weeks (6 months) if they were not vaccinated by outreach services and if they had received the hepatitis B birth dose. Furthermore, the probability of timely completion increased with age and education of mother but decreased by number of siblings. The fit of the overall model in comparison to the null model was assessed (p-value < 0.0001, AUC = 77.9%).

**Table 4:** Binomial generalized linear model showing the factors affecting completion of vaccination by 24 weeks.

Factors included in the final model	OR	95% CI	Estimate	Std. Error	z value	Pr(>|z|)
Intercept	1.59	[0.53–4.74]	0.47	0.56	0.84	0.403
Mother's level of education (Secondary school/University; ref: none/primary education)	1.64	[1.09–2.47]	0.49	0.21	2.39	0.017
Number of siblings (numeric)	0.81	[0.69–0.94]	-0.21	0.08	-2.66	0.008
Age of mother (years) (numeric)	1.05	[1.01–1.09]	0.05	0.02	2.27	0.023
Hepatitis B birth dose (received; ref: not received)	2.14	[1.42–3.24]	0.76	0.21	3.62	<0.001
Place of vaccination (outreach service; ref: vaccinated at the HCF facility)	0.23	[0.14–0.36]	-1.48	0.24	-6.24	<0.001

OR = odds ratio; CI = confidence interval; std = standard; ref = reference; HCF = health care facility

### Hepatitis B birth dose and recall by health facility

At the CHs in Vientiane Capital, 89.7% had received the birth dose according to vaccination card or hospital records compared to 76% in Bolikhamxay (Table 5). All of the parents of participants from Vientiane Capital stated that their children had received the vaccination at birth (BCG and/or hepatitis B birth dose) at the Children’s hospital or another CH. In Bolikhamxay at HC level, only 28.3% of children whose parents remembered that a vaccination was given at birth had record of the hepatitis B birth dose. These participants were almost exclusively born at home.

**Table 5:** Numbers of participants that received the hepatitis B birth dose according to their vaccination cards or vaccination records and the level of health care system.

	Health care level ^a^						
	CH	PH	DH-facility	DH-outreach	HC- facility	HC-outreach	Total N
N received vaccinations at birth according to questionnaire	319	205	266	17	175	92	1074
n received birth dose according to written records	286	195	220	2	139	26	868
(%)	(89.7)	(95.1)	(82.7)	(11.8)	(79.4)	(28.3)	(80.8)

^a^ Participants were grouped according to the place of vaccination with the vaccinations at birth. In the questionnaire, no distinction was made between BCG and hepatitis B birth dose. 88 participants were excluded because participants born outside study, dates not readable, missing, or only in month and year, or parents did not know place of vaccination

CH = central hospitals, PH = Provincial hospital, DH = District hospitals, HC = Health centers, N = total number per group

In Bolikhamxay, both hospital records and vaccination card were available from 479 participants and from those, 313 (65.3%) participants had a vaccination date for the birth dose. The vaccination dates matched in both hospital records and vaccination card for 91.7% of these participants.

The hepatitis B birth dose is recommended to be given within 24 h of birth and latest within the first 7 days after birth [39]. The vast majority of the participants in our study that received the hepatitis B birth dose, did receive it on the day of their birth (93.5%) and only 4.1% did receive it later but within 7 days after the birth date (S6 Table in S1 File).

## Discussion

Reliable and centralized documentation is important for monitoring vaccination coverage and the quality of immunization programmes. In Bolikhamxay, when both hospital records and vaccination card were available, 38% of children had mismatches in at least one of the vaccination date-pairs, and for 63% of those, the date for pentavalent 3 did not match. At the PH level only 2.4% of mismatches were found for the first dose, but in lower ranked health facilities up to 19% of date pairs mismatched. The mismatches increased with each dose (up to 41% at the HC level). There were also 7.1% mismatches in birthdates between the two vaccination records. Mismatches in calendar days, months or years, are indications that dates are not entered simultaneously and at the same session in the vaccination card and hospital records. Vaccination books are usually assigned to each village and taken along to the village during outreach sessions. When the books or the vaccination card are forgotten, retrospective entry of dates may be common. Vaccination dates may also be entered in the vaccination card in advance as a reminder for the parents, but then vaccination may have taken place at another date. Thus, the importance of written documentation needs to be emphasized at every level of the health system. In particular, the management of hospital vaccination books/records needs to be strengthened, the procedures of record keeping in both the vaccination card and the hospital records should be reviewed, and additional training of the health care staff is required to improve the reliability of entries.

Adherence to the vaccination schedule is considered an important quality aspect of routine immunization, even though there is no generally accepted definition of “timely vaccination” [15]. We observed a general delay in vaccination with the pentavalent vaccine, especially in health care settings below the PH level. The difference between median age at vaccination and recommended age at vaccination was highest for pentavalent 3. More than half the children vaccinated at the DH and HC level received all three doses later than recommended. The delay at the DH and HC outreach level may reflect challenges with scheduling outreach visits in those villages. If children miss one visit, they may have to wait for the next visit of the health care workers. In our previous study [20] in this cohort, the place of vaccination was an important predictor for seroprotection. The proportion of protected children was especially low in villages connected to health centers that were located more than one hour of travel time from their district hospital. These findings combined underline the need to strengthen vaccine management in the lower-ranked health care settings. In total, 22% of the children did not complete the immunization series with the pentavalent vaccine by the age of 24 weeks. While we did not find a negative impact of delayed vaccination on seroconversion rates at the CH and PH level, delayed vaccination increases the window of susceptibility and may facilitate disease outbreaks. Since the presence of maternal antibodies may interfere with the infant’s humoral immune response after primary vaccination [40–42], the timing of the first vaccine dose is especially important. However, since the vast majority of the participants in this study were not vaccinated before the scheduled first dose of the pentavalent vaccine at 6 weeks of age, we could not investigate a possible impact of premature vaccination on antibody titers.

In bivariate analyses, essentially all indicators of poor access to health services were very specifically associated with delayed completion of vaccination with the pentavalent vaccine. Since this study included only children who completed vaccination, further studies could investigate whether these are also the risk factors for missing any of the doses of the pentavalent vaccine. After logistic regression analyses, lower education level of the mother, not receiving the hepatitis B birth dose as well as vaccination by outreach service, were independently associated with the delayed completion of full vaccination with the pentavalent vaccine. Due to the geography and infrastructure of the country, around 30% of the villages are classified as remote and are difficult to access. Outreach services should be conducted in at least four rounds per year; however, in 2016, it was estimated that only 9% of remote villages were visited four times [43]. In contrast to our findings, a study conducted in Sri Lanka found higher timeliness in rural areas compared to urban settings [5]. The education of the mother has also been shown in other studies [2, 4, 44] to be an important driver on timely immunization or timely completion of specific vaccinations. These results indicate that there is a need to further improve access to health services, especially in remote rural areas.

Almost all participants in Vientiane Capital but only two thirds of the participants living in Bolikhamxay had received the hepatitis B birth dose; however, this is probably a considerable overestimation of recipients in the general population, since we only enrolled participants with a full course of pentavalent vaccination. Recent data estimate the coverage for the birth dose in 2017 to be 55% nationwide [45]. Even among children who received all three doses of the pentavalent vaccine, only 28.3% had received the hepatitis B birth dose at the health center level in areas covered by outreach. Although the overall number of home births in Lao PDR has decreased from 59% in 2010 [46] to 35% in 2017 [10], these findings are still concerning.

There are several limitations to this study. Those include that the specific place of vaccination (either outreach or on-site) was only available by parents’ recall. Since only children with three doses were included, we cannot determine the number of children who missed one or two doses. Our questionnaire may also not have captured all risk factors for delayed vaccination and our findings may not necessarily be valid for the whole country although it shows a typical pattern.

## Conclusions

During the past few years, major progress has been made in Lao PDR in vaccine coverage and seroconversions rates. In this study, we observed a general delay of vaccination with the pentavalent vaccine and discrepancies in vaccination records. Vaccination delay was associated with indicators of poor access to health services. To further improve the child vaccination programme, reasons for the discrepancies and inconsistency in vaccination documents should be investigated and training of health care staff in robust documentation and management of health records should be provided. We suggest to include timely completion of vaccination as a quality indicator for the national immunization programme in addition to coverage rates and seroconversion rates.

## Supporting information

S1 Fig Timeliness of each dose of the pentavalent vaccine according to the age and the health care levels. A. Timeliness of vaccination with pentavalent 1. B. Timeliness of vaccination with pentavalent 2. C. Timeliness of vaccination with pentavalent 3. Shaded areas indicate the 95% Confidence Interval. Graphs were truncated at 45 weeks to increase visibility. CH = vaccinated at central Hospitals in Vientiane, PH = vaccinated at provincial hospital, DH = vaccinated at district hospital level, HC = vaccinated at health center level. Dashed lines correspond to the median age of vaccination with the pentavalent vaccine. Participants for which the calculated age at vaccination was negative (date of dose before date of birth, indicating a mistake in documentation) were excluded from the graph.
(TIF)

S2 Fig Proportion of participants vaccinated with the third dose of the pentavalent vaccine by health care levels. Mix = participants vaccinated at different health care facilities with one or two of the doses. CH = Central hospital level, DH = District hospital level, HC = health center level. Missing or unreadable dates were excluded from this figure. The age at vaccination was calculated based on the vaccination card, and in case the vaccination card was not available the date in the hospital record was used. The proportion vaccinated at PH, the DH and HC level was compared to the CH level. Data are presented with 95% CI. **** = p<0.0001.
(TIF)

S3 Fig Difference of interval as recommended in schedule and calculated median interval between pentavalent dose 1 and 2 (A) and pentavalent dose 2 and 3 (B) in weeks according to health care level. The intervals were calculated based on the vaccination cards, and in case the vaccination card was not available the date in the hospital records was used. CH = Central hospital level, DH = District hospital level, HC = health center level. Missing or unreadable dates were excluded from this figure.
(TIF)

S1 File (DOCX)
