# Supplementary figures and images for: Timeliness of immunisation with the pentavalent vaccine at different levels of the health care system in the Lao People’s Democratic Republic: A cross-sectional study
Source: PLoS One. 2020 Dec 8;15(12):e0242502. doi: 10.1371/journal.pone.0242502 (PMC7723256; doi:10.1371/journal.pone.0242502)

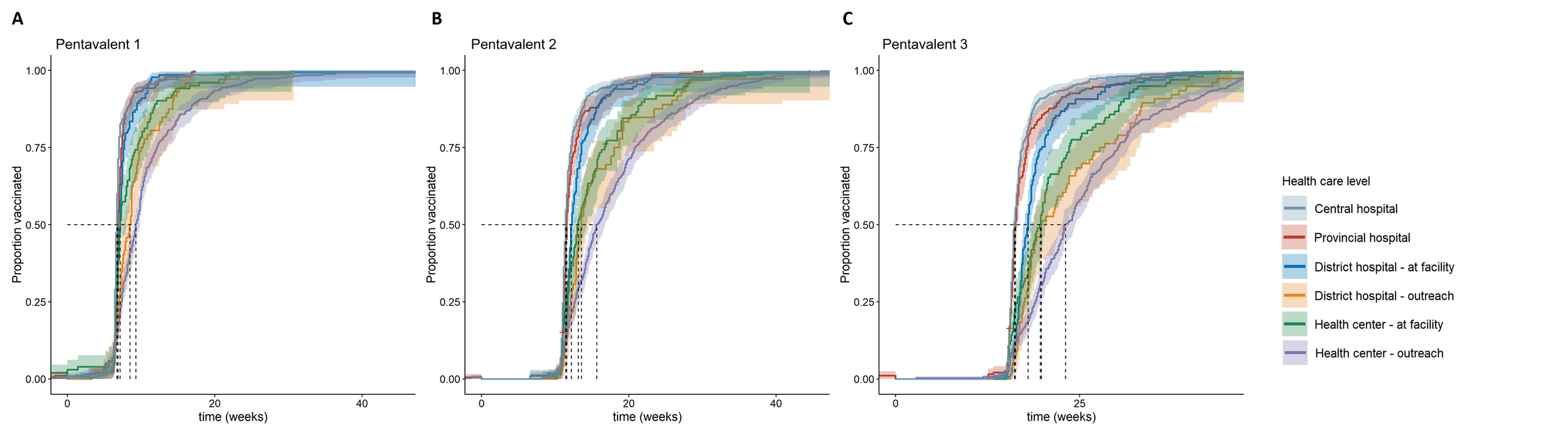

Supplement: S1 Fig — A. Timeliness of vaccination with pentavalent 1. B. Timeliness of vaccination with pentavalent 2. C. Timeliness of vaccination with pentavalent 3. Shaded areas indicate the 95% Confidence Interval. Graphs were truncated at 45 weeks to increase visibility. CH = vaccinated at central Hospitals in Vientiane, PH = vaccinated at provincial hospital, DH = vaccinated at district hospital level, HC = vaccinated at health center level. Dashed lines correspond to the median age of vaccination with the pentavalent vaccine. Participants for which the calculated age at vaccination was negative (date of dose before date of birth, indicating a mistake in documentation) were excluded from the graph. (TIF) [file pone.0242502.s001.tif]

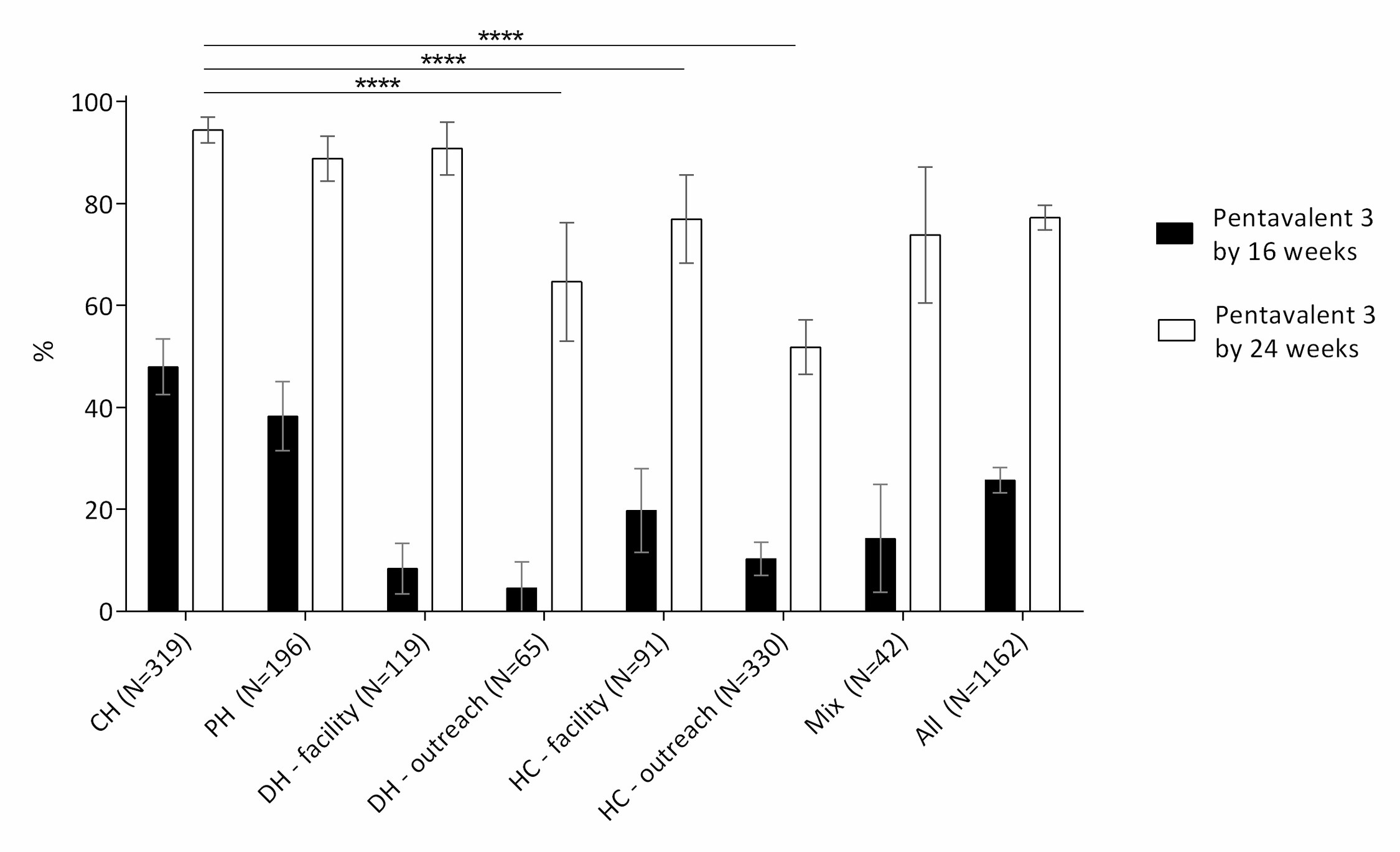

Supplement: S2 Fig — Mix = participants vaccinated at different health care facilities with one or two of the doses. CH = Central hospital level, DH = District hospital level, HC = health center level. Missing or unreadable dates were excluded from this figure. The age at vaccination was calculated based on the vaccination card, and in case the vaccination card was not available the date in the hospital record was used. The proportion vaccinated at PH, the DH and HC level was compared to the CH level. Data are presented with 95% CI. **** = p<0.0001. (TIF) [file pone.0242502.s002.tif]

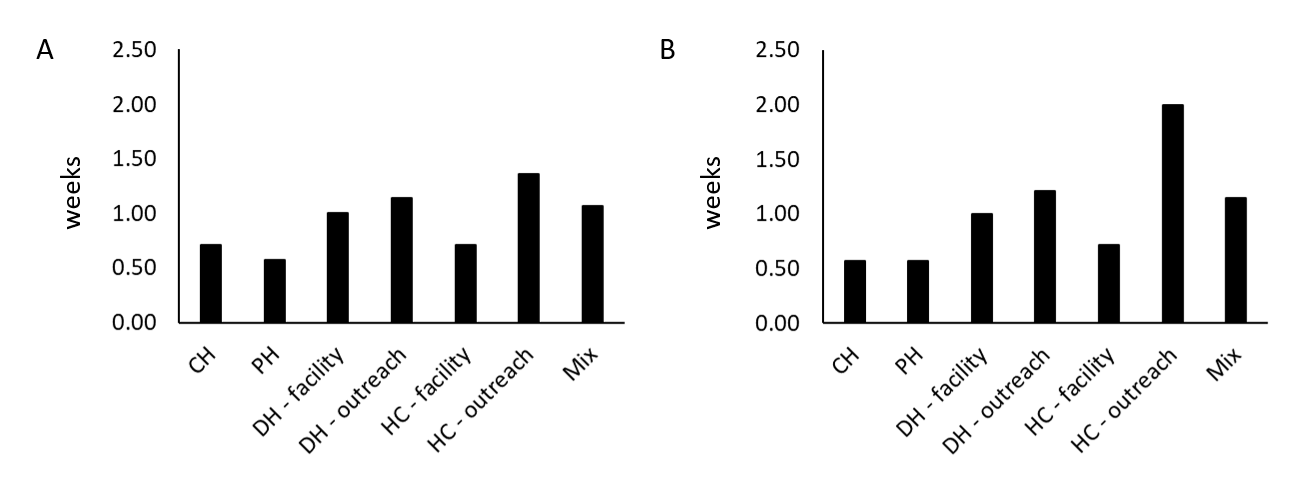

Supplement: S3 Fig — Difference of interval as recommended in schedule and calculated median interval between pentavalent dose 1 and 2 (A) and pentavalent dose 2 and 3 (B) in weeks according to health care level. The intervals were calculated based on the vaccination cards, and in case the vaccination card was not available the date in the hospital records was used. CH = Central hospital level, DH = District hospital level, HC = health center level. Missing or unreadable dates were excluded from this figure. (TIF) [file pone.0242502.s003.tif]
