# Supplementary material for: Timeliness of immunisation with the pentavalent vaccine at different levels of the health care system in the Lao People’s Democratic Republic: A cross-sectional study
Source: PLoS One. 2020 Dec 8;15(12):e0242502. doi: 10.1371/journal.pone.0242502 (PMC7723256; doi:10.1371/journal.pone.0242502)
Supplement: S1 File — (DOCX) [file pone.0242502.s004.docx]

Timeliness of immunisation with the pentavalent vaccine at different levels of the health care system in the Lao People’s Democratic Republic: a cross-sectional study

**Short title** Delayed vaccination in Lao PDR

# Authors

Lisa Hefele^1,2*^; Sengdavanh Syphan^3#a^; Dalouny Xayavong^3#b^; Anousin Homsana^3#c^; Daria Kleine^1,2#d^; Phetsavanh Chanthavilay^3#e^; Phonethipsavanh Nouanthong^1^; Kinnaly Xaydalasouk^1^; Outavong Phathammavong^4^; Somxay Billamay^5^; Anonh Xeuatvongsa^6^; Daniel Reinharz^3,7^; Antony P Black^1^;

Claude P Muller^1,2^

^1^ Lao-Lux Laboratory, Institut Pasteur du Laos, Vientiane, Lao PDR

^2^ Department of Infection and Immunity, Luxembourg Institute of Health, Esch-sur-Alzette, Grand-Duchy of Luxembourg

^3^ Institut de la Francophonie pour la Médecine Tropicale, Vientiane, Lao PDR

^4^ Luxembourg Development Cooperation Agency, Vientiane, Lao PDR

^5^ Children Hospital, Phonetong-Chommany Road, Vientiane, Lao PDR

^6^ Expanded Programme on Immunisation, Vientiane, Lao People’s Democratic Republic

^7^ Département de Médecine sociale et préventive, Université Laval, Québec, Canada

^#a^ Present address: Department of Hygiene and Health Promotion, Ministry of Health, Lao PDR

^#b^ Present address: National Center for Laboratory and Epidemiology, Lao PDR

^#c^ Present address: Lao-Oxford-Mahosot Hospital Wellcome Trust Research Unit, Lao PDR

^#d^ Present address: Saarland University, Homburg, Germany

^#e^ Present address: Institute of Research and Education Development, University of Health Sciences, Vientiane, Lao PDR

## Participants

1174 participants were originally enrolled in the context of an immunogenicity study (Hefele et al., 2019). For this study, 12 participants were excluded from the 1174 participants who were recruited originally, because they were vaccinated at a health care facility outside of the scope of this study or because the vaccination history could not be verified conclusively. In the immunogenicity study, 49 participants were excluded due to the same reasons and because it was not possible to obtain a serum sample or because the serum sample was not sufficient for the laboratory analyses.

## Tables

**S1 Table. Range of the difference between two vaccination dates in days according to health care level and dose.**

|  |  | Difference between mismatches^1^ | | | |
| --- | --- | --- | --- | --- | --- |
|  | Health care level | mean | median | min | max |
| Pentavalent 1 | PH | 1.41 | 0 | 0 | 70 |
|  | DH - facility | 6.72 | 0 | -33 | 315 |
|  | DH - outreach | -2.32 | 0 | -92 | 0 |
|  | HC - facility | 7.46 | 0 | -26 | 372 |
|  | HC - outreach | -2.23 | 0 | -366 | 272 |
| Pentavalent 2 | PH | 0.99 | 0 | 0 | 65 |
|  | DH - facility | -3.11 | 0 | -273 | 77 |
|  | DH - outreach | 1.58 | 0 | -3 | 33 |
|  | HC - facility | -2.88 | 0 | -111 | 41 |
|  | HC - outreach | -6.08 | 0 | -192 | 77 |
| Pentavalent 3 | PH | 1.16 | 0 | -31 | 62 |
|  | DH - facility | -0.02 | 0 | -268 | 113 |
|  | DH - outreach | 0.55 | 0 | -91 | 85 |
|  | HC - facility | -9.92 | 0 | -165 | 42 |
|  | HC - outreach | -9.34 | 0 | -482 | 330 |
| ^1^Participants whose birthdates did not match in the records were excluded | | | | | |
| PH = Provincial hospital, DH = District hospitals, HC = Health centers | | | | | |

**S2 Table. Impact of receiving the pentavalent dose 3 later than 16 weeks on seroconversion.**

| Place of vaccination | Antibody status |  | n age at pentavalent 3 > 16 weeks /  N per group (%) | OR | 95% CI | p-value |
| --- | --- | --- | --- | --- | --- | --- |
| Central hospitals in Vientiane Capital | anti-Tetanus IgG | not protected | 24/44 (54.5) | ref | - | NS |
|  | (N tested = 299) | protected (>0.5 IU/ml) | 159/255 (62.4) | 0.72 | [0.38-1.38] |  |
|  | anti-Diphtheria IgG | not protected | 12/23 (52.2) | ref | - | NS |
|  | (N tested = 301) | protected (≥0.1 IU/ml) | 172/278 (61.9) | 0.67 | [0.29-1.58] |  |
|  | anti-HBs IgG | not protected | 21/38 (55.3) | ref | - | NS |
|  | (N tested = 302) | protected (≥10 IU/ml) | 164/264 (62.1) | 0.75 | [0.38-1.5] |  |
|  | anti-*Haemophilus influenzae* type b IgG | not protected | 2/2 (100) | ref | - | NS |
|  | (N tested = 301) | protected (>1.0 IU/ml) | 182/299 (60.9) | NA | NA |  |
|  | anti-Pertussis IgG | not protected | 108/189 (57.1) | ref | - | NS |
|  | (N tested = 301) | protected (>22 IU/ml) | 76/112 (67.9) | 0.63 | [0.39-1.03] |  |
| Provincial hospital in Bolikhamxay | anti-Tetanus IgG | not protected | 18/33 (54.5) | ref | - | NS |
|  | (N tested = 181) | protected (>0.5 IU/ml) | 106/148 (71.6) | 0.48 | [0.22-1.03] |  |
|  | anti-Diphtheria IgG | not protected | 18/29 (62.1) | ref | - | NS |
|  | (N tested = 181) | protected (≥0.1 IU/ml) | 106/152 (69.7) | 0.71 | [0.31-1.62] |  |
|  | anti-HBs IgG | not protected | 22/37 (59.5) | ref | - | NS |
|  | (N tested = 181) | protected (≥10 IU/ml) | 102/144 (70.8) | 0.60 | [0.29-1.23] |  |
|  | anti-*Haemophilus influenzae* type b IgG | not protected | 33/49 (67.3) | ref | - | NS |
|  | (N tested = 181) | protected (>1.0 IU/ml) | 91/132 (68.9) | 0.93 | [0.46-1.87] |  |
|  | anti-Pertussis IgG | not protected | 85/123 (69.1) | ref | - | NS |
|  | (N tested = 181) | protected (>22 IU/ml) | 39/58 (67.2) | 1.1 | [0.56-2.13] |  |
| CI = Confidence interval, NS = not significant; NA = not available | | | | | | |

**S3 Table. Median interval in weeks between doses according to health care level.**

|  | Interval between vaccinations^1^ | | | | | | | | |
| --- | --- | --- | --- | --- | --- | --- | --- | --- | --- |
|  | Pentavalent 1 to 2 | | | |  | Pentavalent 2 to 3 | |  |  |
| Health care level | N | Median | 4 weeks | >5 weeks |  | N | Median | 4 weeks | >5 weeks |
|  |  | (IQR, in weeks) | % | % |  |  | (IQR, in weeks) | % | % |
| CH | 319 | 4.71 (4.43-5.00) | 64.89 | 33.86 |  | 319 | 4.57 (4.43-5.00) | 69.28 | 30.41 |
| PH | 197 | 4.57 (4.43-5.00) | 64.97 | 29.95 |  | 196 | 4.57 (4.43-5.04) | 62.24 | 30.1 |
| DH - facility | 127 | 5.00 (4.86-5.86) | 23.62 | 72.44 |  | 125 | 5.00 (4.96-6.00) | 23.2 | 74.4 |
| DH - outreach | 68 | 5.14 (4.57-8.07) | 30.88 | 64.71 |  | 69 | 5.21 (4.64-9.82) | 26.09 | 68.12 |
| HC - facility | 97 | 4.71 (4.43-6.29) | 52.58 | 42.27 |  | 93 | 4.71 (4.43-8.04) | 52.69 | 45.16 |
| HC - outreach | 333 | 5.36 (4.43-8.82) | 36.94 | 57.96 |  | 333 | 6.00 (4.43-8.86) | 37.04 | 63.06 |
| Total | 1141 | 4.86 (4.43-5.86) | 49.08 | 47.06 |  | 1135 | 4.86 (4.43-6.14) | 48.63 | 48.28 |
| ^1^The age was calculated with the date written in the vaccination card, if the vaccination card was not present, the date in the hospital records was used; dates that were unreadable, did not exist or where only signature was present, were not included in this table. Children vaccinated at different health care levels were not included. | | | | | | | | | |
| IQR = Interquartile range, CH = Central hospitals, PH = Provincial hospital, DH = District hospitals, HC = Health centers | | | | | | | | | |

**S4 Table. Participants grouped according to timing of intervals between vaccinations with the pentavalent vaccine.**

|  | CH | PH | DH - facility | DH - outreach | HC - facility | HC - outreach | Mix |
| --- | --- | --- | --- | --- | --- | --- | --- |
| Total N | 319 | 196 | 119 | 65 | 91 | 330 | 42 |
| both intervals 4 weeks (%) | 50.47 | 47.96 | 10.92 | 10.77 | 38.46 | 19.39 | 26.19 |
| at least 1 interval longer than 4 weeks (%) | 48.28 | 42.35 | 83.19 | 80.00 | 57.14 | 73.03 | 66.67 |
| Mixed (%) | 1.25 | 9.69 | 5.88 | 9.23 | 4.40 | 7.58 | 7.14 |
| CH = Central hospitals, PH = Provincial hospital, DH = District hospitals, HC = Health centers, N = total numbers of participants vaccinated at the respective health care level | | | | | | | |

**S5 Table. Factors associated with timely completion of primary vaccination with the pentavalent vaccine (in Bolikhamxay province) by 24 weeks.**

| Variables |  | n completed/N per group (%)^1^ | OR | 95% CI | p-value |
| --- | --- | --- | --- | --- | --- |
| Socio-economic Factors |  |  |  |  |  |
| Occupation of mother | Labourer + Farmer+ Trader + Employee + Other | 191/241 (79.25) | 1.00 |  |  |
|  | Housewives | 423/592 (71.45) | 0.66 | [0.46-0.94] | 0.024 |
| Mother's level of education (completed) | None + primary education level | 256/405 (63.21) | 1.00 |  |  |
|  | Secondary school + University | 358/428 (83.64) | 2.98 | [2.15-4.12] | <0.001 |
| Household income (month) | < 1.000.000 Kip | 212/344 (61.63) |  |  |  |
|  | > 1.000.000 Kip | 402/489 (82.21) | 2.88 | [2.09-3.95] | <0.001 |
| Number of siblings | < 2 | 205/267 (76.78) | 1.00 |  |  |
|  | ≥ 2 | 409/566 (72.26) | 0.79 | [0.56-1.11] | 0.178 |
| Number of household members | < 6 | 329/432 (76.16) | 1.00 |  |  |
|  | ≥ 6 | 285/401 (71.07) | 0.77 | [0.56-1.05] | 0.099 |
| Travel time to nearest HCF | < 20 min | 461/580 (79.48) | 1.00 |  |  |
|  | > 20 min | 153/253 (60.47) | 0.39 | [0.29-0.55] | <0.001 |
| Distance to nearest HCF^2^ | < 10 km | 451/608 (74.18) |  |  | NS |
|  | >10 km | 163/225 (72.44) |  |  |  |
| Vaccinee related factors |  |  |  |  |  |
| Ethnicity of parents / guardians | Tai-Kadai | 539/681 (79.15) | 1.00 |  |  |
|  | Hmong-Mien + Mon-Khmer | 75/152 (49.34) | 0.26 | [0.18-0.37] | <0.001 |
| Age of mother | ≤ 30 years | 363/522 (69.54) | 1.00 |  |  |
|  | > 30 years | 199/243 (81.89) | 1.98 | [1.36-2.89] | <0.001 |
| Age of participant | ≤ 12 months | 145/209 (69.38) | 1.00 |  |  |
|  | >12 months | 469/624 (75.16) | 1.34 | [0.95-1.89] | 0.103 |
| Gender of participant | Male | 324/436 (74.31) |  |  | NS |
|  | Female | 290/397 (73.05) |  |  |  |
| Place of birth^3^ | Home | 87/155 (56.13) | 1.00 |  |  |
|  | Health center + District hospital | 308/436 (70.64) | 1.88 | [1.29-2.75] | 0.001 |
|  | Provincial + Central hospital + Other | 219/242 (90.5) | 7.44 | [4.36-12.7] | <0.001 |
| Exclusive breastfeeding | No | 54/73 (73.97) |  |  | NS |
|  | Yes | 560/760 (73.68) |  |  |  |
| Duration exclusive breastfeeding | < 6 months | 416/557 (74.69) |  |  | NS |
|  | ≥ 6 months | 198/276 (71.74) |  |  |  |
| Received antenatal care^4^ | No + I do not know | 41/80 (51.25) | 1.00 |  |  |
|  | Yes | 573/753 (76.1) | 3.03 | [1.89-4.84] | <0.001 |
| TT vaccination during ANC^4^ | No + I do not know | 87/147 (59.18) | 1.00 |  |  |
|  | Yes | 527/686 (76.82) | 2.29 | [1.57-3.32] | <0.001 |
| Number of tetanus doses during ANC^4^ | 0 -3 | 209/326 (64.11) | 1.00 |  |  |
|  | 4-5 | 405/507 (79.88) | 2.22 | [1.62-3.04] | <0.001 |
| Hepatitis B birth dose | Yes | 515/640 (80.47) | 1.00 |  |  |
|  | No | 99/193 (51.3) | 0.26 | [0.18-0.36] | <0.001 |
| Vaccine related factors |  |  |  |  |  |
| District | Paksan | 173/187 (92.51) | 1.00 |  |  |
|  | Khamkheut & Viengthong | 288/464 (62.07) | 0.13 | [0.07-0.24] | <0.001 |
|  | Pakkading | 153/182 (84.07) | 0.43 | [0.22-0.84] | 0.014 |
| Place of vaccination | Vaccinated at Health care facility | 349/384 (90.89) | 1.00 |  |  |
|  | Vaccinated in Outreach | 224/394 (56.85) | 0.13 | [0.09-0.2] | <0.001 |
| ^1^ The date in the vaccination card was used to calculated the age at pentavalent 3. In case the vaccination card was not available, the hospital recrods were used | | | | | |
| ^2^ Distance and travel time to nearest HCF contained similar information, only the variable travel time was included in multivariable analyses | | | | | |
| ^3^ Place of birth and district correlated with place of vaccination, the variables was not included in multivariable analyses | | | | | |
| **^4^** From those variables regarding ANC services, only the main variable of having had ANC or not was included in multivariable analyses | | | | | |
| CI = Confidence interval, NS = not significant; HCF = health care facility; ANC = antenatal care | | | | | |

**S6 Table. The timeliness of receiving the hepatitis B birth dose by health care level.**

|  | Health care level | | | | | | | | |  |
| --- | --- | --- | --- | --- | --- | --- | --- | --- | --- | --- |
|  | CH | PH | DH - facility | DH – outreach | HC - facility | HC - outreach | total | NA^1^ |  | |
| N received birth dose^2^ | 270 | 187 | 206 | 2 | 132 | 24 | 821 | 57 |  | |
| on birth date (%) | 93.0 | 97.9 | 93.2 | 100.0 | 92.4 | 75.0 | 93.5 | 80.7 |  | |
| within 1 week (%) | 5.9 | 1.1 | 4.4 | 0.0 | 3.0 | 12.5 | 4.1 | 19.3 |  | |
| After 7 days (%) | 1.1 | 1.1 | 2.4 | 0.0 | 4.5 | 12.5 | 2.3 | 0.0 |  | |
| ^1^NAs = participants born outside study, dates not readable, missing, or only in month and year, parents did not know place of vaccination | | | | | | | | |  | |
| ^2^Total number does not contain participants whose birth dates did not match in the records, or whose time difference between birth and vaccination was negative | | | | | | | | |  | |
| CH = Central hospitals, PH = Provincial hospital, DH = District hospitals, HC = Health centers | | | | | | | | |  | |

## Figures

**
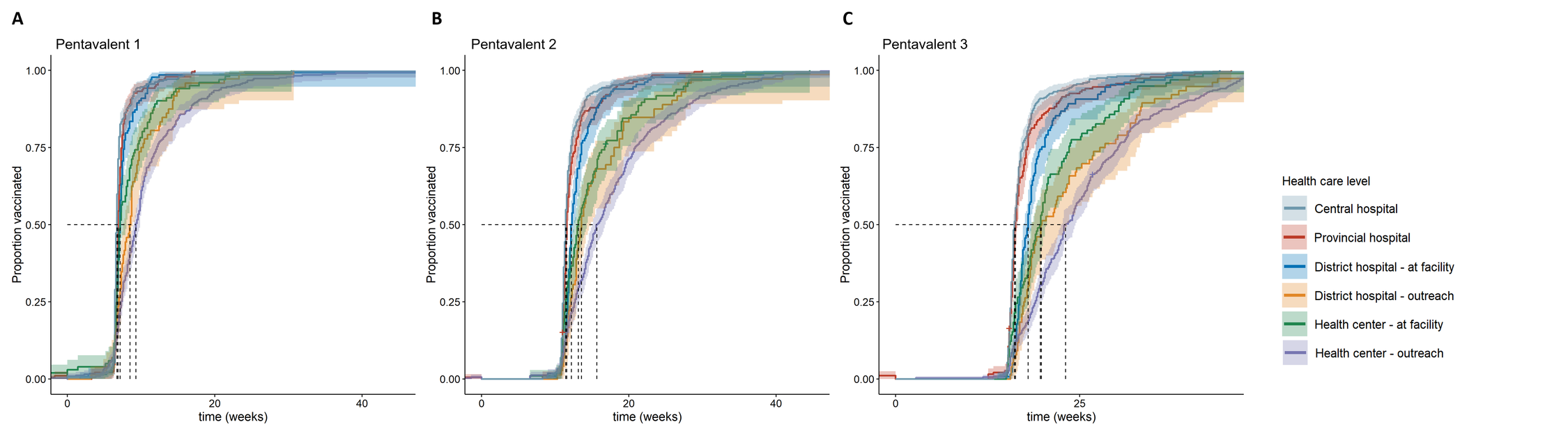
**

**S1 Fig. Timeliness of each dose of the pentavalent vaccine according to the age and the health care levels.** A. Timeliness of vaccination with pentavalent 1**.** B. Timeliness of vaccination with pentavalent 2. C. Timeliness of vaccination with pentavalent 3. Shaded areas indicate the 95% Confidence Interval. Graphs were truncated at 45 weeks to increase visibility. CH = vaccinated at central Hospitals in Vientiane, PH = vaccinated at provincial hospital, DH = vaccinated at district hospital level, HC = vaccinated at health center level. Dashed lines correspond to the median age of vaccination with the pentavalent vaccine. Participants for which the calculated age at vaccination was negative (date of dose before date of birth, indicating a mistake in documentation) were excluded from the graph.


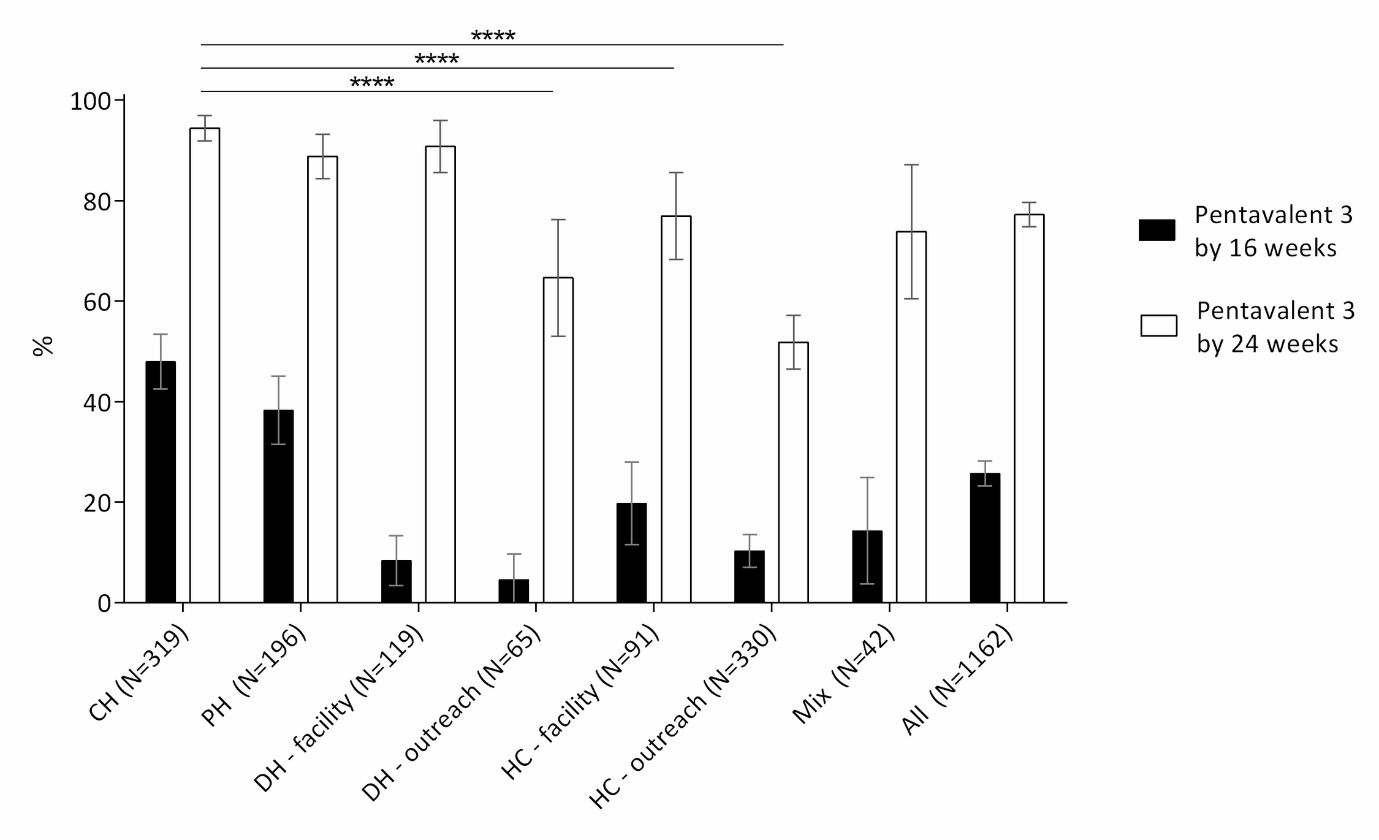


**S2 Fig. Proportion of participants vaccinated with the third dose of the pentavalent vaccine by health care levels.** Mix = participants vaccinated at different health care facilities with one or two of the doses. CH = Central hospital level, DH = District hospital level, HC = health center level. Missing or unreadable dates were excluded from this figure. The age at vaccination was calculated based on the vaccination card, and in case the vaccination card was not available the date in the hospital record was used. The proportion vaccinated at PH, the DH and HC level was compared to the CH level. Data are presented with 95% CI. **** = p<0.0001.





**S3 Fig. Difference of interval as recommended in schedule and calculated median interval between pentavalent dose 1 and 2 (A) and pentavalent dose 2 and 3 (B) in weeks according to health care level.** The intervals were calculated based on the vaccination cards, and in case the vaccination card was not available the date in the hospital records was used. CH = Central hospital level, DH = District hospital level, HC = health center level. Missing or unreadable dates were excluded from this figure.
